# 50 Exercise groups implemented by municipalities promote older adults’ physical functioning

**DOI:** 10.1093/eurpub/ckae114.063

**Published:** 2024-09-26

**Authors:** Eerika Saloranta, Annele Urtamo, Erja Rappe, Satu Jyväkorpi, Pirjo Kalmari, Tuija Ylitörmänen, Hanna Öhman, Katja Borodulin

**Affiliations:** Age Institute, Helsinki, Finland; South-Eastern Finland University of Applied Sciences, Kouvola, Finland; University of Helsinki, Helsinki, Finland; Age Institute, Helsinki, Finland; University of Helsinki, Helsinki, Finland; National Nutrition Council of Finland c/o Finnish Food Authority, Helsinki, Finland; Age Institute, Helsinki, Finland; Finnish Institute for Health and Welfare (THL), Helsinki, Finland; University of Helsinki, Helsinki, Finland; Department of Geriatrics, Helsinki University Hospital, Helsinki, Finland; Age Institute, Helsinki, Finland

## Abstract

**Purpose:**

The implementation and outcomes of physical activity promotion programmes are less studied. This study examines whether physical exercise groups implemented by municipalities are feasible and promote physical functioning among community-dwelling older adults in real-life settings.

**Methods:**

Five municipalities participating in the Strength in Old Age Programme organised guided exercise groups for older adults in Finland. Voluntary participants (mean age 79.6 years (SD 4.2), 74% women) were recruited into strength-balance groups (n = 117) and other exercise groups (n = 34). The strength-balance groups had gym and balance training twice a week. The other groups had chair and balance exercise or senior dance once a week plus home exercise once a week. Short Physical Performance Battery (SPPB) was conducted before and after an 8–12-week exercise period by local exercise instructors. Statistical analyses included changes in SPPB scores and chair stand time within and between exercise groups (t-test) and in subgroup comparisons (baseline SPPB scores <10 points vs. ≥10 points, ANOVA, Dunnett T3).

**Results:**

All exercise groups were implemented as planned, including the SPPB tests by local exercise instructors. Average attendance rate to guided exercise sessions was 91%. Both exercise groups showed significant improvement in the SPPB scores (strength-balance groups: from 9.8 to 11.0 points, p < 0.001; other groups: from 10.4 to 11.2 points, p < 0.001) and chair stand time (strength-balance groups: from 13.7 to 10.9 seconds (s), p < 0.001; other groups: from 12.3 to 10.9 s, p < 0.001). The strength-balance groups had a greater reduction in the chair stand time compared to the other groups (mean change -2.8 s vs. -1.5 s, p < 0.041). The subgroup analyses showed that participants with lower SPPB baseline scores had a greater improvement in the SPPB scores (F(3,147)=34.61, p < 0.001) and chair stand time (F(3,147)=18.30, p < 0.001) compared to the participants with better baseline functioning.

**Conclusions:**

Municipalities can implement physical exercise groups and mobility tests successfully and promote older adults’ physical functioning in real-life settings. Baseline testing is recommended to identify the target groups that might benefit from training the most.

**Funding Source:**

Juho Vainio Foundation, Päivikki and Sakari Sohlberg Foundation

